# Supplementary figures and images for: Multiparametric Approach to the Colorectal Cancer Phenotypes Integrating Morphofunctional Assessment and Computer Tomography
Source: Cancers (Basel). 2024 Oct 15;16(20):3493. doi: 10.3390/cancers16203493 (PMC11506564; doi:10.3390/cancers16203493)

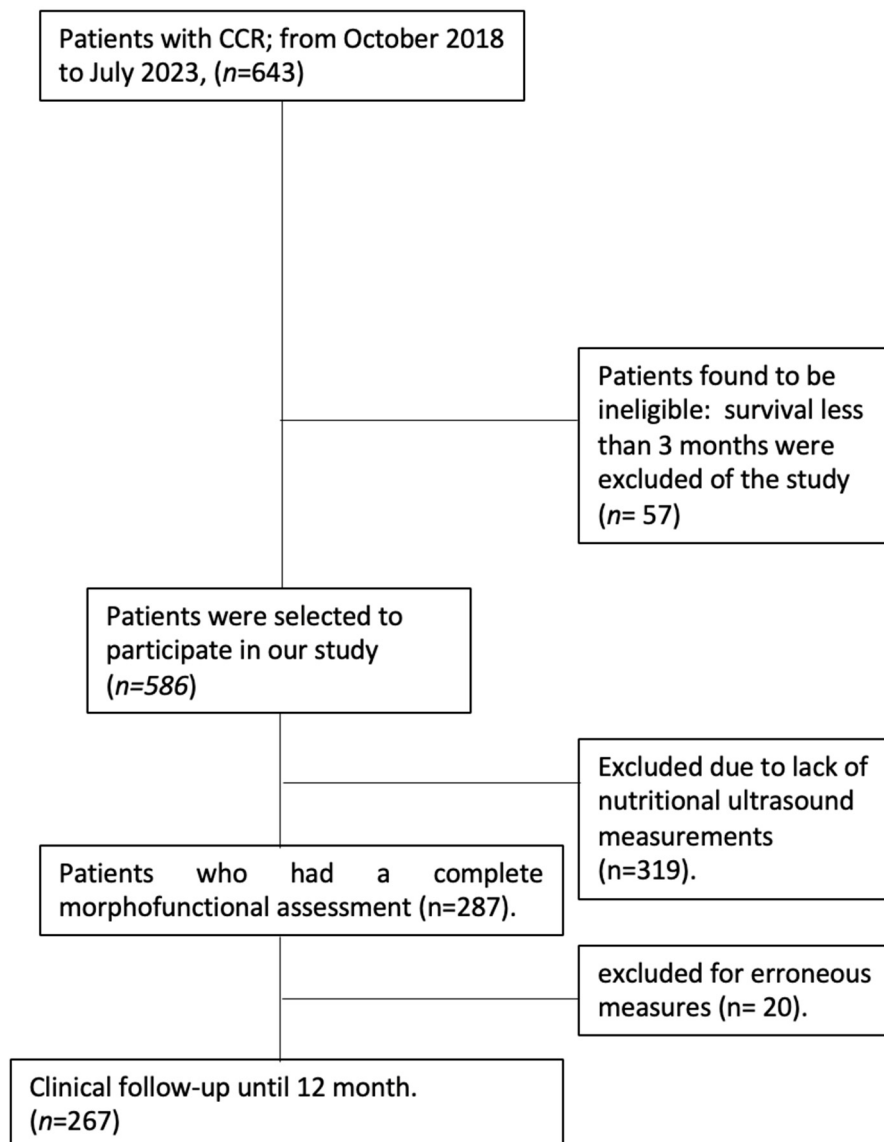

**Supplementary Materials:** Figure S1, Flow chart.

Supplement: Supplementary file 1 [file cancers-16-03493-s001.zip › cancers-3212615-supplementary.pdf]
